# Effects of a lifestyle intervention on body composition in prostate cancer patients on androgen deprivation therapy

**DOI:** 10.1002/crt2.13

**Published:** 2020-05-26

**Authors:** Zachary L. Chaplow, Brian C. Focht, Alexander R. Lucas, Elizabeth Grainger, Christina Simpson, Jackie Buell, Ciaran M. Fairman, Jennifer M. Thomas-Ahner, Jessica Bowman, Victoria R. DeScenza, J. Paul Monk, Amir Mortazavi, Steven K. Clinton

**Affiliations:** 1 Kinesiology, Department of Human Sciences, The Ohio State University, 305 Annie and John Glenn Ave, Columbus, OH 43210, USA; 2 Department of Health Behavior and Policy, Virginia Commonwealth University, Richmond, VA, USA; 3 Comprehensive Cancer Center, The Ohio State University, Columbus, OH 43210, USA; 4 Department of Internal Medicine, Division of Medical Oncology, The Ohio State University, Columbus, OH 43210, USA; 5 Medical Dietetics, School of Health and Rehabilitation Sciences, The Ohio State University, Columbus, OH 43210, USA; 6 Exercise Medicine Research Institute, Edith Cowan University, Joondalup, WA, Australia

**Keywords:** Androgen deprivation, Hormone therapy, Body composition, Prostate cancer, Exercise, Diet

## Abstract

**Background:**

Exercise and dietary (EX+D) interventions could represent an optimal treatment for attenuating or reversing adverse effects of androgen deprivation therapy (ADT) in prostate cancer (PCa) patients. The Individualized Diet and Exercise Adherence-Pilot (IDEA-P) trial compared the effects of an EX+D intervention relative to standard-of-care (SC) treatment among PCa patients undergoing ADT. The present study evaluated the effects of the EX+D intervention on body composition (BC) obtained via dual-energy X-ray absorptiometry (DXA) in a subsample of IDEA-P patients. A secondary objective was to explore the association of adiposity and lean mass with mobility performance and strength.

**Methods:**

Complete DXA data were acquired from a subsample of 22 PCa patients (EX+D: *n* = 13; SC: *n* = 9) at baseline and 3 month follow-up. Intention-to-treat analysis included data from 30 participants (*M* age = 66.28; SD = 7.79) with baseline DXA assessments.

**Results:**

Intention-to-treat analysis revealed EX+D resulted in significant improvements in fat mass (*P* = 0.022), per cent fat mass (*P* = 0.028), trunk fat mass (*P* = 0.017), fat mass/lean mass (*P* = 0.040), and per cent lean mass (*P* = 0.026) vs. SC. EX+D also resulted in more favourable changes in appendicular lean mass/body mass (*d* = 0.59). Select BC outcomes were also significantly correlated with mobility performance and strength (*P* < 0.05) at 3 month follow-up.

**Conclusions:**

Findings suggest the EX+D intervention resulted in superior preservation of lean tissue and improvement in adiposity relative to SC treatment. Results underscore the utility of implementing EX+D interventions for preserving muscle mass and reducing adiposity in PCa patients undergoing ADT.

## Introduction

The efficacy of androgen deprivation therapy (ADT) in adjuvant and neo-adjuvant treatment of prostate cancer (PCa) patients is well established.^1,2^ However, prolonged ADT consistently results in declines in muscle mass, muscle strength, and bone mineral density as well as concomitant increases in fat mass.^3–7^ The combination of sarcopenia (age-related decreased muscle mass and function) and obesity (excess adiposity), commonly defined as sarcopenic obesity, yields a synergistic effect that heightens risk for adverse health outcomes among PCa patients including functional decline and chronic conditions such as metabolic syndrome, type II diabetes, and cardiovascular disease.^8–11^ Given the emerging interest in delineating the extent to which changes in body composition may impact cancer care and survivorship,^12^ together with the established influence of ADT on muscle mass and adiposity, it is becoming increasingly important to identify efficacious supportive care interventions that can effectively attenuate or reverse the adverse effects of ADT upon body composition outcomes among PCa patients.

In this regard, there is growing evidence that exercise interventions can offset adverse changes in body composition observed with ADT. Recent findings suggest resistance exercise is effective at preserving^4,13–16^ and even increasing^17^ muscle mass, strength and function in PCa patients undergoing ADT. However, extant evidence presently demonstrates that resistance exercise alone does not consistently yield meaningful reductions in body fat percentage or total adiposity.^13,14^ This is a notable limitation, as lean and fat mass have exhibited independent effects on mobility and differences in association with health outcomes in older adults. Findings from multiple trials support the implication of fat mass as a critical factor to consider when evaluating prevalence of sarcopenia and mobility-related outcomes.^18,19^ Relative lean mass measures accounting for body weight and fat mass, such as appendicular lean mass (ALM) relative to total body weight, have shown strong associations with risk factors for cardiometabolic diseases.^20^ Furthermore, total fat mass to lean mass ratios have been shown to be significant predictors of physical limitation, metabolic syndrome, and cardiovascular risk in older adults.^21–23^ To date, measures of lean mass accounting for adiposity have received limited attention in men on ADT.^24^ Brown *et al.*^12^ recently proposed that, although the majority of oncology studies exploring the prognostic value of body composition have focused on changes in lean mass outcomes, the development of multi-modal interventions with the potential to preserve and/or increase muscle mass while concomitantly decreasing adiposity is of critical importance for cancer patients and survivors who may be at heightened risk for sarcopenic obesity and its associated adverse health outcomes.^12^

Consistent with this position, it is well established within the behavioural weight management literature that lifestyle interventions that combine modification in exercise participation and dietary intake result in superior improvements in weight management outcomes relative to either exercise or dietary intervention alone.^25–28^ Behavioural weight management interventions combining exercise and dietary (EX+D) approaches to produce intentional weight loss that maximize fat mass loss while also preserving the greatest amount of muscle mass are linked with the most favourable improvements in physical function and chronic disease risk factors in aging adults.^29^ Emerging evidence from multiple small-scale lifestyle intervention trials demonstrate that lifestyle interventions combining EX+D intake components resulted in meaningful improvements in clinically relevant physical function, fitness, and health outcomes among PCa patients undergoing ADT.^30–33^ Although these findings illustrate the potential synergistic benefit of combining EX+D interventions in the supportive care of PCa patients on ADT, reliance upon body mass index and waist circumference assessment in these prior investigations limit what can be concluded about the effects of such multi-modal lifestyle interventions upon changes in adiposity and muscle that are associated with key clinical and health outcomes.^12^ Accordingly, the effect of combined EX+D interventions upon change in whole-body and regional lean mass and fat mass outcomes among PCa patients undergoing ADT has yet to be systematically investigated and warrants further inquiry.

Findings from our recently completed Individualized Diet and Exercise Adherence-Pilot (IDEA-P) trial demonstrate that a lifestyle intervention combining EX+D intake components yielded significant, clinically meaningful improvements in exercise participation, self-reported dietary intake, mobility performance, muscular strength, and select body composition outcomes relative to standard-of-care (SC) treatment in PCa patients undergoing ADT.^34^ Specifically, observed changes in body composition, assessed using air displacement plethysmography, revealed the lifestyle intervention resulted in significant reductions in body fat percentage and fat mass while fat-free mass remained stable at 3 month follow-up. In addition to the air displacement plethysmography measure completed by all study participants, a subsample of IDEA-P trial participants also completed a body composition assessment obtained via dual-energy X-ray absorptiometry (DXA). Accordingly, this subsample from the IDEA-P trial provides a unique opportunity to examine the potentially synergistic effects of a lifestyle intervention combining change in both EX+D behaviours upon key lean mass and adiposity outcomes among PCa patients undergoing ADT. Therefore, the objectives of the present investigation are to (i) examine changes in lean mass and adiposity outcomes following the lifestyle and SC interventions and (ii) explore the extent to which lean mass and adiposity are related to mobility performance and muscular strength in the subsample of PCa patients who completed DXA body composition assessments in the IDEA-P trial. It was hypothesized that the lifestyle intervention would result in superior improvements in lean mass and adiposity outcomes relative to SC and that more favourable lean mass and fat mass would be associated with enhanced mobility performance and muscular strength.

## Methods

### Study design and trial participants

We performed ancillary analysis of body composition subsample data collected as part of the IDEA-P trial, which was a single-blind, two-arm randomized controlled pilot trial. Study support was provided by National Cancer Institute Grant #R03 CA16296901 (Trial Registration: NCT02050906 Registered; 24 January 2014). Detailed reports of the study design, trial procedures, and interventions have been published previously.^34–36^ A brief summary of the relevant methods and procedures is provided here. A total of 32 PCa patients on ADT participated in the IDEA-P trial. The participants were randomly assigned to either the EX+D (*n* = 16) or SC (*n* = 16) treatment arm following the completion of the baseline screening visit. At follow-up visits, assessments of all outcomes were obtained by study staff blinded to participants’ treatment group assignments. Of the 32 total participants in the IDEA-P trial, complete DXA data were obtained from 22 participants (EX+D: *n* = 13; SC: *n* = 9). Intention-to-treat analysis, using all available baseline data, resulted in a total of 30 participants included in the present analysis (EX+D: *n* = 14; SC: *n* = 16). Demographic characteristics, recruitment, attrition, and adherence rates of the total sample cohort have also been reported previously.^34,36^ Participant characteristics including average age, time on ADT (in months since commencement), body height, body mass index with classification (normal weight, overweight, and obese), and baseline values for all body composition outcomes are provided in Table 1.

### Procedures

#### Exercise and dietary intervention

The EX+D intervention involved a multi-component approach designed to facilitate EX+D behaviour change and promote adherence, independent of study staff, to changes in lifestyle behaviour across the 3 month intervention. The personalized exercise component integrated 1 h supervised exercise sessions performed twice per week and involved a combination of resistance and aerobic exercise. Participants were encouraged to gradually increase independent activity and decrease sedentary time in order to progress towards accruing a total weekly volume of physical activity consistent with national guidelines for health and well-being.^37^ The integrated dietary component included eight group-based nutritional counselling sessions with a registered dietitian completed immediately following exercise sessions and two individualized phone sessions. The dietary component was designed to be consistent with the nutritional objectives recommended by 2010–2015 Dietary Guidelines for Americans,^38^ the American Heart Association/American College of Cardiology,^39^ and the World Cancer Research Fund/American Institute for Cancer Research (WCRF/AICR)^40^ and aimed to provide basic nutrition education/counselling to all participants, address contemporary topics in nutrition and cancer, and personalized guidance towards adopting changes in dietary intake characterized by shifts towards a diet rich in whole grains, vegetables, and fruits; limited consumption of processed high fat, low nutrient dense foods; reduced intake of red and processed meats; and overall caloric intake levels that promote achieving and maintaining a healthy body weight and avoiding weight gain.^36^ The purpose of the group-mediated cognitive behavioural counselling component was to facilitate the development, practice, and mastery of self-regulatory skills necessary to adopt and maintain change in EX+D behaviour.

#### Standard-of-care intervention

Men randomized to the SC treatment arm received usual PCa treatment and standard disease management education, as well as additional educational literature describing the WCRF/AICR dietary and physical activity guidelines and education.^40^ Participants received bi-weekly 20 min phone contacts delivered by study staff focusing on routine aspects of PCa self-management.^34–36^

### Measures

#### Body composition

Previously published body composition outcomes obtained from all participants enrolled in the IDEA-P trial were measured using air displacement plethysmography.^34^ The present study focuses on the subsample of trial participants who also completed a DXA (Lunar iDXA, GE Healthcare, Madison, WI, USA) assessment. The DXA scans were used to analyse whole-body and regional body composition outcomes. DXA has well-established validity and reliability and remains the gold standard for clinical assessment of body composition.^41^

For the purposes of the present study, body composition outcomes were reported as whole-body and/or regional measures. Whole-body measures included body mass, fat mass, per cent fat mass, lean mass, per cent lean mass, and the proportion of fat mass to lean mass. Regional measures included appendicular fat mass, trunk fat mass, gynoid fat mass, ALM, ALM/height squared, and the proportion of ALM to body mass. Appendicular fat mass was calculated as the sum of fat mass in arms and legs. ALM was calculated as the sum of lean mass in arms and legs, assuming all tissues other than fat and bone mineral content are skeletal muscle.

#### Relationship between body composition and mobility performance and muscular strength

Changes in the IDEA-P trial’s primary outcomes of mobility performance, assessed using the 400 m walk test (400MWT), and muscular strength, assessed using leg extension one repetition maximum (1RM), have been reported previously.^34^ These measures of mobility performance and muscular strength have well-established validity and reliability and are highly predictive of subsequent mobility disability and mortality.^42^ The 400MWT and leg extension 1RM measures were included in the present study to evaluate the extent to which adiposity and lean mass values are associated with mobility and strength outcomes at 3 month follow-up, which represents the independent maintenance phase of behaviour change, in the IDEA-P trial.

### Statistical analysis

Data were analysed using separate univariate analyses of covariance (ANCOVAs) examining the effect of treatment group on change in body composition observed from baseline to 3 month assessment. In each model, change from baseline was used as the outcome variable. Time on ADT (in months) and baseline value of each outcome measure were included in the models as covariates. ANCOVAs were conducted using the intention-to-treat principle to account for missing data and the last-observation-carried-forward approach, used to impute change across time as zero. Statistical tests were two-tailed with an alpha level of 0.05 required for statistical significance. All analyses were conducted using SPSS 23.0 (IBM SPSS Statistics for Windows, Version 23, Armonk, NY; IBM Corp.). Additionally, estimates of effect size (Cohen’s *d*) were calculated by dividing the adjusted group mean differences by the pooled standard deviation to determine the magnitude of differences observed for each outcome.^43^ Finally, given that the lifestyle intervention was specifically designed to foster improvements in mobility performance and muscular strength, partial correlation analyses controlling for age and time on ADT were conducted to examine the relationship between select body composition outcomes and mobility performance and muscular strength at the 3 month follow-up assessment.

## Results

### Patient characteristics

Participant characteristics are presented in Table 1. The EX+D and SC groups are balanced with no significant differences between groups at baseline.

### Whole-body measures

After time on ADT and baseline values were controlled, ANCOVA of change yielded a significant treatment main effect on body mass (*F*(_1, 26_) = 4.82, *P* = 0.037; *d* = −0.78), fat mass (*F*(_1, 26_) = 5.98, *P* = 0.022; *d* = −0.86), per cent fat mass (*F*(_1, 26_) = 5.44, *P* = 0.028; *d* = −0.79), per cent lean mass (*F*(_1, 26_) = 5.59, *P* = 0.026; *d* = 0.79), and the proportion of fat mass to lean mass (*F*(_1, 26_) = 4.68, *P* = 0.040; *d* = −0.71) all favouring the EX+D intervention. Participants in the EX+D group lost 2.50 kg of body mass across the 3 month trial. Approximately 75% of the 2.50 kg body mass loss observed in the EX+D group was attributable to loss of fat mass alone. In contrast, the SC treatment resulted in a significantly smaller loss of body mass (0.54 kg) and a 0.08 kg increase in fat mass at 3 month follow-up. Additionally, the EX+D intervention yielded a >1% decrease from baseline fat mass percentage, while SC was associated with a modest (0.22%) increase in per cent fat mass at 3 months.

Although both groups lost a modest amount of lean mass overall (EX+D: −0.49 kg; SC: −0.67 kg), there were no statistically significant treatment differences in whole-body lean mass change observed at 3 month follow-up *(P* = 0.683). However, the amount of lean mass retained favoured the EX+D intervention group as participants lost a smaller amount of absolute lean mass according to adjusted mean differences (*d* = 0.25). SC yielded a modest decrease in per cent lean mass change, while the EX+D intervention resulted in a >1% increase at 3 months (*d* = 0.79). Collectively, the reduction of fat mass and preservation of lean mass observed within the EX+D intervention group resulted in statistically significant (*P* = 0.040) differences in the total proportion of fat mass to lean mass change, with a value < 1.0 indicating a more favourable change in the fat to lean ratio (EX+D: −0.03; SC: 0.01; *d* = −0.71). Results for change in whole-body composition are presented in Table 2.

### Regional measures

ANCOVA of change across the regional body composition measures yielded significant treatment main effects on trunk fat mass (*F*(_1, 26_) = 6.45, *P* = 0.017; *d* = −0.95) and gynoid fat mass (*F*(_1, 26_) = 4.39, *P* = 0.046; *d* = −0.70) favouring the EX+D intervention. Participants in the EX+D group lost approximately 1.26 kg of trunk fat mass, whereas SC resulted in a 0.11 kg increase at 3 month follow-up. Similarly, the EX+D intervention resulted in a 0.28 kg decrease in gynoid fat mass at 3 months, while SC remained approximately unchanged (−0.03 kg). Although group differences in appendicular fat mass change did not reach statistical significance (EX+D: −0.57 kg; SC: −0.06 kg; *P* = 0.132), a medium effect size was observed favouring the EX+D intervention (*d* = −0.56). ANCOVA of change revealed no statistically significant differences in ALM (*F*_(1, 26)_ = 0.09, *P* = 0.765; *d* = 0.11), ALM/height^2^ (*F*_(1, 26)_ = 0.01, *P* = 0.922; *d* = 0.03) or ALM/body mass (*F*_(1, 26)_ = 2.63, *P* = 0.117; *d* = 0.59) between groups at 3 month follow-up. However, superior relative ALM change was observed favouring the EX+D intervention. The EX+D intervention group gained approximately 0.5% ALM/body mass, while the SC treatment resulted in a small decrease in proportional ALM change (*d* = 0.59). Results for change in regional body composition are presented in Table 3.

### Correlation analyses

Partial correlation analyses controlling for age and time on ADT were conducted to examine the relationships between both the adiposity and lean mass outcomes and mobility performance (400MWT) and muscular strength (leg extension 1RM) at 3 months. Results revealed that fat mass (*r* = 0.45; *P* = 0.017), per cent fat mass (*r* = 0.57; *P* = 0.007), per cent lean mass (*r* = −0.58; *P* = 0.001), fat mass/lean mass (*r* = 0.56; *P* = 0.002), appendicular fat mass (*r* = 0.51; *P* = 0.006), trunk fat mass (*r* = 0.38; *P* = 0.047), gynoid fat mass (*r* = 0.49; *P* = 0.009), and ALM/body mass (*r* = −0.62; *P* < 0.001) were significantly correlated with improved mobility performance at 3 months. Additionally, lean mass (*r* = 0.67; *P* < 0.001), ALM (*r* = 0.70; *P* < 0.001), ALM/height^2^ (*r* = 0.52; *P* = 0.004), and ALM/body mass (*r* = 0.42; *P* = 0.028) were significantly correlated with greater muscular strength at 3 months. Collectively, the partial correlation analyses suggest that body composition outcomes were associated with more favourable levels in mobility performance and muscular strength at the 3 month follow-up, which represents the independent maintenance phase of behaviour change, in the IDEA-P trial. Significant partial correlations for mobility performance and muscular strength are presented in Tables 4 and 5, respectively.

## Discussion

Findings from the present study revealed that a lifestyle intervention combining personalized EX+D intake prescriptions resulted in significant improvements in adiposity and lean mass outcomes relative to SC treatment in a subsample of PCa patients undergoing ADT in the IDEA-P trial. Specifically, the lifestyle intervention, when compared with SC treatment, yielded meaningful differences in changes in body mass, fat mass, per cent fat mass, per cent lean mass, and ratio of fat mass to lean mass. Thus, IDEA-P is one of the first studies to demonstrate that a lifestyle intervention designed to produce intentional weight loss in overweight or obese patients by combining modification in exercise participation and dietary intake results in meaningful reductions in adiposity while largely preserving lean mass yielding favourable change in the proportion of whole-body lean mass relative to fat mass and regional lean mass relative to body size. Additionally, changes in fat mass and lean mass were associated with superior mobility performance and muscular strength, indicating that the observed body composition changes may be integral to attenuating or reversing the adverse effects of ADT that contribute to frailty and functional decline. The unique pattern of associations observed between fat mass and mobility performance and muscle mass and muscular strength is consistent with emerging evidence from recent lifestyle weight management intervention trials among older adults, suggesting change in fat mass is strongly related to mobility performance while preserving lean mass is vital for maintaining or increasing muscular strength^44–46^ and extend these findings to PCa patients undergoing ADT.

To date, the majority of lifestyle intervention research targeting PCa patients has focused upon the effects of resistance exercise. Findings from the extant research demonstrate that resistance exercise reliably preserves or increases muscle mass but does not consistently elicit meaningful reductions in adiposity.^13–17^ Conversely, combining change in exercise participation and dietary intake in the present investigation yielded significant reductions in whole-body and regional adiposity outcomes. Given that prior intervention studies in PCa patients have focused on the importance of prognostic effects of muscle,^12^ the observed change in adiposity is a novel finding that may have important implications for integrating lifestyle EX+D intervention in supportive PCa care. In evaluating the potential research and clinical significance of the observed reduction in fat mass outcomes, it is critical to recognize that three patients in the EX+D intervention arm were not overweight and received personalized EX+D intake prescriptions intended to keep them weight stable. However, the majority of PCa patients in IDEA-P were overweight or obese. Accordingly, the primary objective of the personalized EX+D intake prescription for these patients was to foster modest intentional weight loss that would aid in achieving a healthier body weight characterized by simultaneously reducing adiposity and preserving as much muscle mass as possible.

In interpreting the potential clinical utility of lifestyle weight management interventions for PCa patients, it is important to recognize the benefit of weight loss for overweight or obese cancer patients remains disputed and presently unclear.^12,47^ As anticipated with lifestyle interventions designed to produce gradual intentional weight loss, while the EX+D intervention resulted in a significant decline in fat mass, it was also accompanied by a 0.49 kg decline in lean mass. The loss of lean mass following the lifestyle intervention was not significantly different from the 0.67 kg reduction in lean mass observed with SC. Nonetheless, owing to concerns that intentional weight loss may exacerbate muscle wasting, sarcopenia, risk of frailty, and functional decline associated with ADT, questions of the clinical benefit of integrating this lifestyle intervention approach in the treatment of older PCa patients may emerge given the persisting debate of the safety and efficacy of weight loss interventions for obese cancer patients.^12^ In evaluating the reduction in lean mass observed in the present trial, it is important to recognize that PCa patients in the lifestyle intervention lost a four-fold greater amount of fat mass (1.75 kg) relative to lean mass lost (0.40 kg), and this proportion is consistent with ratios observed in prior lifestyle weight management intervention studies in older adults.^29,48^ It is also notable that increases in per cent whole-body lean mass relative to body mass (1.02%) and the ratio of ALM to body mass (0.47%) were observed following the lifestyle intervention. Furthermore, the adiposity and lean mass outcomes were associated with superior mobility performance and muscular strength at 3 month follow-up. While the present lifestyle intervention focused upon personalized changes in dietary intake involving modest caloric restriction for overweight or obese patients, it is possible that providing additional nutritional support within the context of these dietary changes, such as increased protein intake or creatine supplementation,^49–51^ could augment the preservation of muscle mass without compromising the goal of intentional weight loss and systematic evaluation of these approaches warrants further inquiry.

The present findings suggest that exercise and diet interventions producing intentional weight loss driven by substantial reduction in fat mass that yield a more favourable proportion of lean mass to body mass, relative to pre-intervention values, are linked to beneficial changes in mobility performance and muscular strength. Thus, it is possible that concerns of intentional weight loss heightening risk of frailty and functional decline due to the associated loss of lean mass may be mitigated, in part, if the weight loss is characterized by substantial fat mass loss and modest lean mass loss resulting in a net increase in the overall proportion of muscle mass to body mass retained. However, the extent to which lifestyle intervention-induced changes in absolute lean mass and adiposity vs. change in the proportion of lean mass to adiposity may be of more prognostic value for determining clinically relevant health and functional outcomes among obese PCa patients on ADT has yet to be adequately delineated and warrants future inquiry.

The unique patterns of association observed in the correlation analyses demonstrated that adiposity outcomes were more strongly related to mobility performance while the lean mass outcomes were more consistently associated with muscular strength. These findings add to a growing body of evidence suggesting that fat mass loss may be primarily responsible for weight loss associated improvements in mobility.^29,45^ Nonetheless, this is one of the first lifestyle EX+D intervention trials to explore these associations in PCa patients on ADT, and replication of this pattern in large-scale randomized trials is required to confirm the veracity of the associations observed in the present pilot trial.

Strengths of the present study include being one of the first investigations to implement a theoretically driven, evidence-based lifestyle intervention combining change in EX+D intake in PCa patients undergoing ADT. Additionally, IDEA-P is one of the first to use DXA-acquired assessments, the gold standard in body composition assessment, to evaluate changes in lean mass and adiposity outcomes following an intervention integrating modification of both exercise participation and dietary intake components. Although the present findings are novel and contribute to a more comprehensive understanding of the benefits of lifestyle interventions for PCa on ADT, there are select limitations that should be considered when interpreting the present results. For example, the small sample size of the present pilot trial does not provide adequate statistical power to detect meaningful differences in all relevant outcomes. Furthermore, although an intention-to-treat analysis was performed, the last-value-carried-forward technique has well-established limitations and is a quite conservative approach to addressing missing data. However, with the small sample size, observed effect size estimates, and amount of missing data, alternative less-biased imputation procedures are not likely to result in meaningful increases in the accuracy of estimation of imputation values. Clearly, large-scale optimally powered randomized controlled lifestyle intervention trials incorporating more sophisticated maximum likelihood imputation methods are warranted in future inquiry. Finally, although DXA-acquired lean mass and adiposity assessments are the gold-standard measure of clinically determined body composition, it does not provide a method for direct assessment of visceral fat or change in fat infiltration, which have been identified as important mechanisms underlying change in mobility performance and chronic disease risk with lifestyle weight management interventions.^29,46^ These are outcomes that should be explored in future investigations in PCa patients on ADT.

## Conclusions

Findings from the present study revealed (i) that a lifestyle intervention combining personalized EX+D intake prescriptions resulted in significant improvements in adiposity and lean mass outcomes relative to SC treatment in a subsample of PCa patients undergoing ADT in the IDEA-P trial and (ii) that changes in fat mass and lean mass were associated with superior mobility performance and muscular strength. Taken collectively, the present findings suggest a lifestyle EX+D intervention that produced intentional weight loss, characterized by augmented fat loss and preservation of lean mass for overweight and obese patients, may be integral to attenuating or reversing the adverse effects of ADT that contribute to frailty and functional decline. These findings underscore the synergistic benefits of lifestyle interventions integrating personalized EX+D prescription in the supportive care of PCa patients undergoing ADT.

## Figures and Tables

**Table 1:** Baseline participant characteristics.

Measure	EX+D	SC	*p* value
Sample size, *n* (%)	14 (46.7)	16 (53.3)	
Age (years)	67.9 ± 7.9	64.3 ± 6.1	0.202
Time on ADT (months)	26.0 ± 25.5	20.4 ± 21.0	0.514
Height (cm)	180.4 ± 5.5	178.3 ± 5.4	0.288
BMI (kg/m^2^)	29.0 ± 4.4	31.4 ± 5.9	0.225
BMI classification, *n* (%)			
Normal	3 (10.0)	0 (0.0)	
Overweight	5 (16.6)	8 (26.7)	
Obese	6 (20.0)	8 (26.7)	
Whole-body measures			
Body mass (kg)	94.6 ± 15.6	99.4 ± 15.4	0.413
Fat mass (kg)	34.5 ± 9.7	38.5 ± 12.0	0.331
Fat mass (%)	36.0 ± 5.0	38.1 ± 5.8	0.302
Lean mass (kg)	57.0 ± 7.5	57.9 ± 5.6	0.727
Lean mass (%)	60.7 ± 4.8	58.8 ± 5.5	0.327
FM/LM	0.60 ± 0.14	0.66 ± 0.17	0.309
Regional measures			
AFM (kg)	13.7 ± 3.8	14.5 ± 5.2	0.627
Trunk fat mass (kg)	19.8 ± 6.1	23.0 ± 7.5	0.220
Gynoid fat mass (kg)	5.1 ± 1.6	5.8 ± 2.1	0.371
ALM (kg)	27.0 ± 4.2	27.1 ± 3.2	0.927
ALM/ht^2^ (kg/m^2^)	8.3 ± 1.1	8.5 ± 1.0	0.505
ALM/BM (%)	28.6 ± 2.2	27.5 ± 2.7	0.227

Data presented as *M* ± SD or *n* (%).

ADT, androgen deprivation therapy; AFM, appendicular fat mass; ALM, appendicular lean mass; BM, body mass; BMI, body mass index; EX+D, exercise and dietary; FM, fat mass; ht^2^, height squared; LM, lean mass; SC, standard of care; SD, standard deviation.

**Table 2:** Adjusted mean changes in whole-body composition measures.

Measure	3 month change				*p* value	*d*
	EX+D		SC			

	Mean	SD	Mean	SD		
Body mass (kg)	−2.50	2.91	−0.54	2.07	0.037*	−0.78
Fat mass (kg)	−1.89	2.75	0.08	1.70	0.022*	−0.86
Fat mass (%)	−1.06	1.93	0.22	1.25	0.028*	−0.79
Lean mass (kg)	−0.49	1.50	−0.67	1.29	0.683	0.13
Lean mass (%)	1.02	1.84	−0.22	1.24	0.026*	0.79
FM/LM	−0.03	0.05	0.01	0.04	0.040*	−0.71

EX+D, exercise and dietary; FM, fat mass; LM, lean mass; SC standard of care; SD standard deviation.

* Time on ADT and baseline value adjusted mean change significantly different from SC (*P* < 0.05).

**Table 3:** Adjusted mean changes in regional body composition measures.

Measure	3 month change				*p* value	*d*
	EX+D		SC			

	Mean	SD	Mean	SD		
AFM (kg)	−0.57	1.33	−0.06	0.68	0.132	−0.48
Trunk fat mass (kg)	−1.26	1.68	0.11	1.13	0.017*	−0.95
Gynoid fat mass (kg)	−0.28	0.45	−0.03	0.23	0.046*	−0.70
ALM (kg)	−0.19	1.14	−0.31	0.90	0.765	0.11
ALM/ht^2^ (kg/m^2^)	−0.07	0.35	–0.08	0.29	0.922	0.03
ALM/BM (%)	0.47	0.99	−0.02	0.64	0.117	0.59

AFM, appendicular fat mass; ALM, appendicular lean mass; BM, body mass; EX+D, exercise and dietary; ht^2^, height squared; SC, standard of care; SD, standard deviation.

* Time on ADT and baseline value adjusted mean change significantly different from SC (*P* < 0.05).

**Table 4:** Significant partial correlations with 400 m walk time at 3 months.

Measure	*r* value	*p* value
Whole-body measures		
Fat mass (kg)	0.45	0.017
Fat mass (%)	0.57	0.007
Lean mass (%)	−0.58	0.001
FM/LM	0.56	0.002
Regional measures		
AFM (kg)	0.51	0.006
Trunk fat mass (kg)	0.38	0.047
Gynoid fat mass (kg)	0.49	0.009
ALM/BM (%)	−0.62	0.000

Age and time on ADT adjusted significant partial correlation (*P* < 0.05).

AFM, appendicular fat mass; ALM, appendicular lean mass; BM, body mass; FM, fat mass; LM, lean mass.

**Table 5:** Significant partial correlations with leg extension 1RM at 3 months.

Measure	*r* value	*p* value
Whole-body measures		
Lean mass (kg)	0.67	0.000
Regional measures		
ALM (kg)	0.70	0.000
ALM/ht^2^ (kg/m^2^)	0.52	0.004
ALM/BM (%)	0.42	0.028

Age and time on ADT adjusted significant partial correlation (*P* < 0.05).

1RM, one repetition maximum; ALM, appendicular lean mass; BM, body mass; ht^2^, height squared.
